# Multi-Dimensional Behavioral Signature Analysis for Video Bullet Comment Steganography Detection

**DOI:** 10.3390/e28090999

**Published:** 2026-09-07

**Authors:** Yitong Liu, Hongwei Zhao

**Affiliations:** 1 School of Software Engineering, Beijing Jiaotong University, Beijing 100044, China; yitongliu768@gmail.com; 2 Key Laboratory of Big Data and Artificial Intelligence in Transportation, Ministry of Education, Network and Information Technology Center, Beijing Jiaotong University, Beijing 100044, China

**Keywords:** video-sharing platforms, bullet comment, covert channel, behavioral signature analysis, detection framework

## Abstract

The proliferation of bullet comment systems on video-sharing platforms has created novel opportunities for covert communication. Recent research has demonstrated multiple bullet comment-based steganographic paradigms: time modulation, time attribute shifting, live broadcast game-based channels, and generative stegotext via frame comments. This paper presents Multi-Dimensional Behavioral Signature Analysis (MDBSA), a unified detection framework integrating seven behavioral dimensions to systematically characterize anomalies from four documented bullet comment steganographic paradigms. We construct a synthetic benchmark spanning six video categories with controlled steganographic injection; extract features capturing temporal, spatial, content, and structural patterns; and evaluate it using GroupKFold cross-validation to prevent data leakage from overlapping sliding windows. On this synthetic benchmark, MDBSA features combined with Gradient Boosting achieve 96.9% accuracy (F1 = 96.8%, AUC = 0.996), compared with 65.6% for logistic regression on the same features. Per-paradigm detection rates on the benchmark range from 98.4% to 100.0%.

## 1. Introduction

Covert communication conceals information within innocuous covers  [1,2]. Traditional steganography exploits redundancy in images [3,4,5], audio [6], and network protocols [7,8]. Deep learning methods such as HiDDeN [9] and SteganoGAN [10] further advanced high-capacity embedding. The emergence of interactive social media has introduced behavioral covert channels that manipulate interaction patterns rather than cover content [11,12].

Video bullet comment systems (Bilibili, Niconico, iQIYI, YOUKU) support real-time floating comments overlaid on video with billions of daily interactions. Their millisecond-precision timestamps, accessible attribute logs, and high natural variance in sending frequency enable sophisticated covert channels.

Mao et al. [13] pioneered bullet comment steganography via time modulation, followed by time attribute shifting [14] (2.5 times per-comment payload increase), game-based CovLBCG [15], and generative steganography [16] using ResNet-LSTM on frame comments. Related text steganography methods [17,18,19] modify linguistic content rather than interaction metadata, further motivating behavioral-level detection.

Existing detection approaches—including statistical tests [20], ensemble classifiers [21], and deep learning steganalysis [22,23]—were not designed for the full spectrum of contemporary behavioral attacks [24]. The ε-similarity and compressibility score methods [25] effectively detect timing anomalies but are limited against content and generative paradigms. A comprehensive survey by Li et al. [11] emphasizes the gap between covert channel construction and multi-modal detection in social media environments.

This paper addresses the detection gap through three contributions. First, we propose MDBSA, a unified framework that formalizes seven behavioral dimensions, each targeting a specific steganographic paradigm. Unlike prior work that analyzes timing alone, MDBSA integrates temporal regularity, time-shift deviation, spatial distribution, content entropy, game-instruction patterns, generative text signatures, and length-interval correlation anomalies into a single detection architecture. Second, we provide a reproducible evaluation using a synthetic benchmark spanning six video categories and the four documented attack paradigms considered in this study, with GroupKFold cross-validated results and open release of the dataset generation methodology. Third, we conduct a systematic empirical analysis comparing MDBSA features against baseline representations (ε-similarity, raw statistics) to quantify the contribution of multi-dimensional behavioral profiling, and examine feature group interactions through ablation studies.

The novelty of this work relative to existing bullet comment steganalysis is threefold. (i) Unified multi-dimensional coverage: To our knowledge, MDBSA is the first detection framework that jointly addresses all four published bullet comment steganographic paradigms (time modulation, time attribute shifting, game-based channels, and generative stegotext) in a single architecture; prior detectors operate on timing statistics only [25] and cannot capture content- or position-based attacks. (ii) Interpretable behavioral signature representation: Instead of feeding raw statistics to a black-box classifier, MDBSA derives seven per-dimension anomaly signatures, each with a documented interpretation and a traceable link to a specific attack vector, which supports both detection and post hoc analysis of why a stream is flagged. (iii) Quantified added value over the dominant character-composition features: The ablation analysis explicitly quantifies how much of the detection performance the multi-dimensional framework contributes beyond the simple emoji/digit-ratio features, rather than treating feature-group interactions as a black box. These three aspects jointly constitute the claimed novelty, and Section 4.2, Section 4.3, Section 4.4 and Section 4.5 provide direct experimental support for each of them, as summarized in the Conclusion.

## 2. Related Work

This section reviews the four documented bullet comment steganographic paradigms and existing detection approaches. We first describe each attack vector—time modulation, time attribute shifting, game-based covert channels, and generative stegotext—to establish the threat model. We then survey current detection methods and identify gaps that motivate the unified multi-dimensional detection framework proposed in Section 3.

### 2.1. Bullet Comment Steganography Methods

Time Modulation [13]. Mao et al. analyzed 5.3 million bullet comments from six platforms. Their method embeds secrets through a dual-index timestamp mechanism where the number of stego comments *n* is encoded in the interval between the last two timestamps ($n=t_{N}-t_{N-1}+t_{l}$), and position indices are determined by adjacent intervals ($l_{i}=t_{i+1}-t_{i}$). ChaCha20 encryption protects index data.

Time Attribute Shifting [14]. This method modulates both relative and absolute time as deviations from reference comments, with coupling $\tau_{i}^{a}=\gamma \cdot \tau_{i}^{r}+\omega_{i}^{\prime }$ where γ≈2/3. Security analysis employs ε-similarity score and compressibility score [25], with SVM-based AUC evaluation for parameter selection.

Game-Based CovLBCG [15]. Exploits live broadcast bullet comment games (e.g., Plants vs. Zombies), where formatted game commands embed arithmetic-encoded payloads. The LBCG channel has high interaction frequency (median 74 s interval vs. 30,745 s for Weibo).

Generative CVF [16]. Liu et al. generate stego-comments via ResNet-LSTM rather than modifying existing ones. Secrets are represented as vocabulary indices and transformed through pseudorandom W-d matrices into frame index sequences with strictly increasing output and minimum spacing *d*. Distracting words are interspersed to obscure message length.

### 2.2. Detection Methods

Traditional steganalysis (Chi-square, KS-test, entropy analysis) achieves limited accuracy due to high natural bullet comment variance. Feature-engineered ML (SVM, Random Forest) improves detection but may not generalize across attack types.

Zillien and Wendzel [25] proposed ε-similarity and compressibility scores for timing anomaly detection. The ε-similarity score quantifies normalized inter-arrival deviations as $\varepsilon =\mathrm{median}\{|\tau_{i+1}-\tau_{i}|/\tau_{i}\}$; steganographic encoding produces larger, more structured deviations yielding elevated ε. The compressibility score estimates Kolmogorov-complexity-like regularity, where hidden information reduces randomness.

However, both metrics are limited to timing-domain analysis. They cannot capture content-level manipulation (generative stegotext [16], emoji/digit ratio distortion), spatial coordination patterns (synchronized vertical positioning), and their threshold calibration is unreliable in high-variance bullet comment environments where bursty normal traffic mimics steganographic patterns. These limitations motivate our multi-dimensional approach.

## 3. Proposed Method: MDBSA

This section formalizes the MDBSA detection framework: overall architecture (Section 3.1), behavioral dimensions and signature computation (Section 3.2), sliding window feature extraction (Section 3.3), dimension-to-feature mapping (Section 3.4), and the Behavioral Anomaly Score (Section 3.5).

### 3.1. Framework Overview

**Figure 1:**
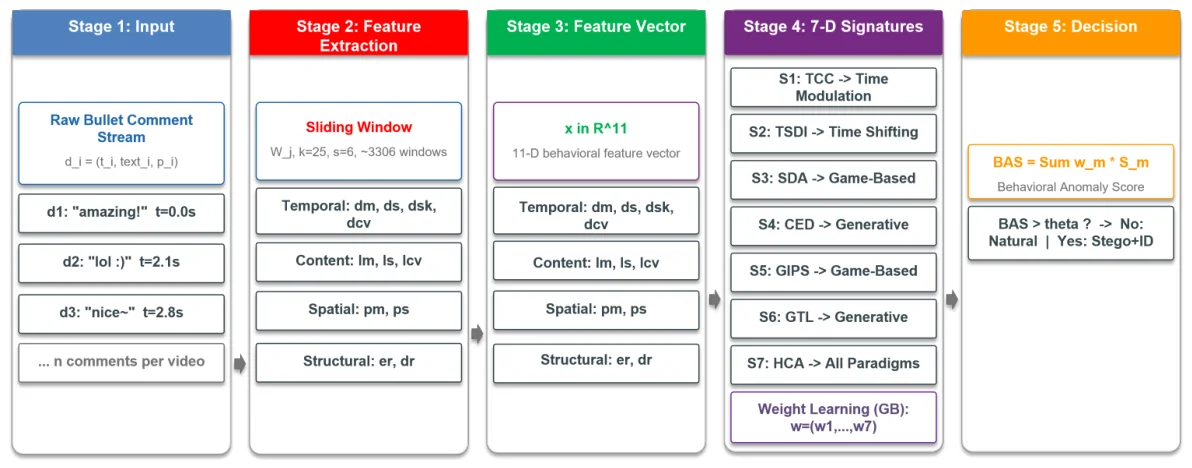
MDBSA framework: Sliding windows extract four feature groups (temporal, spatial, content, structural) → 11 features → seven signatures $\left\{S_{1},\ldots ,S_{7}\right\}$→ weighted BAS for detection.

The MDBSA framework is M=(B,F,S,W,D), as shown in Figure 1, where B are seven behavioral observation sequences, F maps to $x\in R^{11}$, $S=\{S_{m}\}$ computes $S_{m}:R^{11}\to \left[0,1\right]$, W is a learned weight vector ($\sum w_{m}=1$, $w_{m}\geq 0$), and $D:R^{7}\to \left\{0,1\right\}\times \left\{1,\ldots ,4\right\}$ outputs a binary label and paradigm identifier (the present study evaluates only binary detection).

### 3.2. Behavioral Dimension Formalization

We define seven signature computation modules $S_{1}$ through $S_{7}$, each producing a normalized anomaly score in [0,1]. Each module operates on a dimension-specific subset of the 11 extracted features (defined in Section 3.3) and compares observed values against category-specific baseline statistics (μ,σ) estimated from natural (non-stego) bullet comment sequences.

Normalization procedure. All seven signatures are normalized to [0,1] as follows. The raw statistics (dcv, pm, ps, lm, ls, lcv, er, dr, $\varepsilon_{r}$, $\varepsilon_{a}$) are first z-score-standardized with category-specific parameters $\left(\mu_{z,c},\sigma_{z,c}\right)$: $\tilde{x}=\left(x-\mu_{z,c}\right)/\sigma_{z,c}$. Each signature is then computed from the standardized quantities via the formulas in Equations (1)–(8) and finally clipped to [0,1] ($S_{2}$ via min-max rescaling, $S_{4}$ and $S_{7}$ via clipping). Crucially, all baseline statistics (μ,σ) and all normalization parameters $\left(\mu_{z,c},\sigma_{z,c}\right)$ are estimated exclusively from the training folds of each GroupKFold split (Algorithm 1), so that no information from validation or test windows enters any normalization step; the estimated parameters are stored and applied unchanged to validation and test windows at evaluation time.
**Algorithm 1** MDBSA Training**Input:** Labeled sequences $\left\{D^{\left(i\right)}\right\}$ with labels $\left\{y^{\left(i\right)}\right\}$, window size k=25, stride s=6, category labels $\left\{c^{\left(i\right)}\right\}$**Output:** $w^{*}$, $\theta^{*}$, baselines $\left\{\left(\mu_{c},\sigma_{c}\right)\right\}$, normalization params $\left\{\left(\mu_{z,c},\sigma_{z,c}\right)\right\}$  1:Partition each $D^{\left(i\right)}$ into windows $\left\{W_{j}\right\}$ with (k,s)  2:Split into K=5 GroupKFold folds (sequence-level separation)  3:**for** each fold **do**  4:      Estimate $\left(\mu_{c},\sigma_{c}\right)$ per category from *training-fold* normal-only ($y^{\left(i\right)}=0$) windows  5:      Compute $x_{j}\in R^{11}$ (Equations (10)–(20)) and $S_{j}\in {\left[0,1\right]}^{7}$ (Equations (1)–(8)) for train/val/test windows  6:      Fit $\left(\mu_{z,c},\sigma_{z,c}\right)$ on *training-fold* windows per category; normalize train/val/test with these parameters  7:      Learn w via softmax-param. logistic regression on training $\left\{S_{j}\right\}$  8:      Select $\theta^{*}$ maximizing Youden’s index on validation windows (step 0.01)  9:      Evaluate on test-fold windows10:**end for**11:$w^{*}\leftarrow \mathrm{mean}\left(w_{\mathrm{folds}}\right)$, $\theta^{*}\leftarrow \mathrm{mean}\left(\theta_{\mathrm{folds}}\right)$

$S_{1}$: Temporal Regularity Deviation (TRD). We quantify inter-arrival regularity via the coefficient of variation dcv=ds/dm (Equation (13)). Natural bullet comment streams exhibit Poisson-like patterns with a characteristic dcv range; time modulation attacks [13] impose deterministic structure that reduces dcv. $S_{1}$ measures the deviation of dcv from its category-specific baseline:
  (1) $$S_{1}=\min\left(\frac{|\mathrm{dcv}-\mu_{\mathrm{dcv}}|}{\sigma_{\mathrm{dcv}}},\;1\right)$$
  where $\mu_{\mathrm{dcv}}$ and $\sigma_{\mathrm{dcv}}$ are the mean and standard deviation of dcv estimated from natural (non-stego) bullet comment sequences of the same video category. The min-with-1 cap normalizes $S_{1}$ to [0,1]. $S_{1}$ targets the time modulation paradigm.
$S_{2}$: Time-Shift Deviation Index (TSDI). Since the detector observes only the timestamp sequence $\left\{t_{1},\ldots ,t_{n}\right\}$ (not the attacker’s reference offsets), we construct observable proxies: local interval residuals $\delta_{i}=\Delta t_{i}-\mathrm{median}\left(\Delta t_{i-2:i+2}\right)$ and normalized second differences $\gamma_{i}=\left|\Delta t_{i+1}-\Delta t_{i}\right|/\left(\Delta t_{i}+\epsilon \right)$. ε-similarity scores [25] on these proxies:
  (2) $$\varepsilon_{r}=\mathrm{median}\left\{\frac{|\delta_{i+1}-\delta_{i}|}{\delta_{i}+\epsilon }\right\},\;\varepsilon_{a}=\mathrm{median}\left\{\frac{|\gamma_{i+1}-\gamma_{i}|}{\gamma_{i}+\epsilon }\right\}$$
  where $\epsilon =10^{-3}$ prevents division by zero. Let $\left(\mu_{\varepsilon_{r}},\mu_{\varepsilon_{a}}\right)$ and $\Sigma_{\varepsilon }$ be the mean vector and covariance matrix of $\left(\varepsilon_{r},\varepsilon_{a}\right)$ estimated from natural bullet comment. $S_{2}$ is the Mahalanobis distance:
  (3) $$S_{2}=\sqrt{{\left(\varepsilon -\mu_{\varepsilon }\right)}^{⊤}\Sigma_{\varepsilon }^{-1}\left(\varepsilon -\mu_{\varepsilon }\right)}$$
  normalized to [0,1] via min-max scaling. $S_{2}$ targets the time attribute shifting paradigm using only the observable timestamp sequence, without requiring knowledge of the attacker’s reference structure.
$S_{3}$: Spatial Distribution Anomaly (SDA). Game-based steganography [15] encodes commands at specific vertical positions, creating detectable spatial regularities. Let pm and ps be the sliding window position mean and standard deviation (Equations (17) and (18)). Given category-specific baselines $\left(\mu_{\mathrm{pm}},\sigma_{\mathrm{pm}}\right)$ and $\left(\mu_{\mathrm{ps}},\sigma_{\mathrm{ps}}\right)$,
  (4) $$S_{3}=\frac{1}{2}\left[\left|\frac{\mathrm{pm}-\mu_{\mathrm{pm}}}{\sigma_{\mathrm{pm}}}\right|+\left|\frac{\mathrm{ps}-\mu_{\mathrm{ps}}}{\sigma_{\mathrm{ps}}}\right|\right]$$
  clipped to [0,1]. Elevated $S_{3}$ indicates that comment positions deviate systematically from the expected distribution. $S_{3}$ targets the game-based paradigm.
$S_{4}$: Content Entropy Deviation (CED). Generative stegotext [16] produces comments with length distributions distinct from human-written text. Let lm, ls, lcv be the window-level length statistics (Equations (14)–(16)) and H(l)=−∑p(l)logp(l) be the empirical entropy of comment lengths within the window. $S_{4}$ is the normalized entropy deviation:
  (5) $$S_{4}=\frac{|H\left(l\right)-{\bar{H}}_{\mathrm{cat}}|}{\sigma_{H}}$$
  where ${\bar{H}}_{\mathrm{cat}}$ and $\sigma_{H}$ are the mean and standard deviation of length entropy for the video category, estimated from natural sequences. $S_{4}$ is clipped to [0,1]. $S_{4}$ targets the generative paradigm.
$S_{5}$: Game-Instruction Pattern Score (GIPS). CovLBCG commands [15] have rigid formats with high digit ratios. $S_{5}$ captures digit prevalence and positional regularity:
  (6) $$S_{5}=\mathrm{dr}\cdot \left(1-\frac{\min(|\tilde{\mathrm{pm}}-{\tilde{p}}_{\mathrm{game}}|,{\tilde{\Delta }}_{p})}{{\tilde{\Delta }}_{p}}\right)$$
  where $\tilde{\mathrm{pm}}=\mathrm{pm}/H$ is the normalized mean vertical position (relative to video player height *H*), dr is the window digit ratio (Equation (20)), ${\tilde{p}}_{\mathrm{game}}\approx 0.25$ is the normalized modal game-command position (estimated from training data as ${\tilde{p}}_{\mathrm{game}}=\arg\max_{\tilde{p}}\;p\left(\tilde{p}|\mathrm{game}\right)$), and ${\tilde{\Delta }}_{p}=0.15$ is the normalized position tolerance. The normalization *H* makes the feature resolution-independent across platforms. $S_{5}\in \left[0,1\right]$; higher values indicate windows with concentrated digit usage at game-typical screen positions. $S_{5}$ targets the game-based paradigm.
$S_{6}$: Generative Text Likelihood (GTL). $S_{6}$ combines length coefficient-of-variation and emoji-ratio anomalies:
  (7) $$S_{6}=\frac{1}{2}\left[\left|\frac{\mathrm{lcv}-\mu_{\mathrm{lcv}}}{\sigma_{\mathrm{lcv}}}\right|+\left|\frac{\mathrm{er}-\mu_{\mathrm{er}}}{\sigma_{\mathrm{er}}}\right|\right]$$
  clipped to [0,1]. $\mu_{\mathrm{lcv}},\sigma_{\mathrm{lcv}},\mu_{\mathrm{er}}$, and $\sigma_{\mathrm{er}}$ are category-specific baseline statistics of length coefficient-of-variation and emoji ratio, respectively. $S_{6}$ targets the generative paradigm.
$S_{7}$: Length-Interval Correlation Anomaly (LICA). In natural bullet comment streams, a weak negative correlation typically exists between inter-arrival times and comment lengths: frequent short comments during high-activity periods and less frequent longer comments during sparse periods. Steganographic injection may perturb this natural relationship by imposing independent encoding on timing and content dimensions. $S_{7}$ measures the absolute deviation of the within-window Pearson correlation from its category-specific baseline:
  (8) $$S_{7}=\left|\;\rho_{\mathrm{window}}\left(\Delta t,l\right)-{\bar{\rho }}_{\mathrm{cat}}\;\right|$$
  where $\rho_{\mathrm{window}}$ is the Pearson correlation between $\Delta t_{i}$ and $l_{i}$ within the current window, and ${\bar{\rho }}_{\mathrm{cat}}$ is the mean correlation for the video category. $S_{7}$ is clipped to [0,1]. Unlike the single-dimension signatures $S_{1}$–$S_{6}$, $S_{7}$ captures cross-dimensional dependencies between timing and content, making it applicable as a complementary signal across all paradigms.

### 3.3. Feature Extraction

Let a bullet comment $d_{i}=\left(t_{i},l_{i},p_{i},e_{i},c_{i}\right)$ where $t_{i}$ is the timestamp, $l_{i}$ the length, $p_{i}$ the vertical position, $e_{i}\in \left\{0,1\right\}$ emoji presence, and $c_{i}\in \left\{0,1\right\}$ digit presence. A sliding window $W_{j}$ (k=25, s=6) extracts

(9) $$W_{j}=\left\{d_{1+(j-1)s},\;\ldots ,\;d_{k+(j-1)s}\right\},\;j=1,\ldots ,\left\lfloor \frac{n-k}{s}\right\rfloor +1$$

Inter-arrival times are $\Delta t_{i}=t_{i+1}-t_{i}$. Each window yields 11 features in four groups, capturing temporal, content, spatial, and structural patterns [26]:

Temporal features (capturing regularity of sending intervals):
  (10) $${\mathrm{dm}}_{j}=\frac{1}{k-1}\sum_{i=1}^{k-1}\Delta t_{i}\;\;\;\;\;\;\;\;$$
  (11) $${\mathrm{ds}}_{j}=\sqrt{\frac{1}{k-2}\sum_{i=1}^{k-1}{\left(\Delta t_{i}-{\mathrm{dm}}_{j}\right)}^{2}}\;$$
  (12) $${\mathrm{dsk}}_{j}=\frac{\frac{1}{k-1}\sum_{i=1}^{k-1}{\left(\Delta t_{i}-{\mathrm{dm}}_{j}\right)}^{3}}{{\left({\mathrm{ds}}_{j}\right)}^{3}}\;$$
  (13) $${\mathrm{dcv}}_{j}=\frac{{\mathrm{ds}}_{j}}{{\mathrm{dm}}_{j}}\;\;\;\;\;\;\;\;$$
  where ${\mathrm{dm}}_{j}$ is the mean inter-arrival time, ${\mathrm{ds}}_{j}$ the standard deviation, ${\mathrm{dsk}}_{j}$ the skewness, and ${\mathrm{dcv}}_{j}={\mathrm{ds}}_{j}/{\mathrm{dm}}_{j}$ the coefficient of variation for scale-invariant comparison.
Content features (capturing comment length patterns):
  (14) $${\mathrm{lm}}_{j}=\frac{1}{k}\sum_{i=1}^{k}l_{i}\;\;\;\;\;\;\;\;\;$$
  (15) $${\mathrm{ls}}_{j}=\sqrt{\frac{1}{k-1}\sum_{i=1}^{k}{\left(l_{i}-{\mathrm{lm}}_{j}\right)}^{2}}$$
  (16) $${\mathrm{lcv}}_{j}=\frac{{\mathrm{ls}}_{j}}{{\mathrm{lm}}_{j}}\;\;\;\;\;\;\;\;$$
  where ${\mathrm{lm}}_{j}$ is the mean comment length, ${\mathrm{ls}}_{j}$ the standard deviation, and ${\mathrm{lcv}}_{j}={\mathrm{ls}}_{j}/{\mathrm{lm}}_{j}$ the coefficient of variation (elevated in generative stegotext with mixed-length content).
Spatial features (capturing vertical positioning regularity):
  (17) $${\mathrm{pm}}_{j}=\frac{1}{k}\sum_{i=1}^{k}p_{i}\;\;\;\;\;\;\;\;$$
  (18) $${\mathrm{ps}}_{j}=\sqrt{\frac{1}{k-1}\sum_{i=1}^{k}{\left(p_{i}-{\mathrm{pm}}_{j}\right)}^{2}}$$
  where $p_{i}\in \left[0,H\right]$ is the vertical coordinate (pixels), ${\mathrm{pm}}_{j}$ the mean position, and ${\mathrm{ps}}_{j}$ the positional spread. Natural positions are diffuse; game commands concentrate at specific screen regions.
Structural features (capturing character composition anomalies):
  (19) $${\mathrm{er}}_{j}=\frac{1}{k}\sum_{i=1}^{k}e_{i}$$
  (20) $${\mathrm{dr}}_{j}=\frac{1}{k}\sum_{i=1}^{k}c_{i}$$
  where $e_{i}\in \left\{0,1\right\}$ indicates whether comment *i* contains at least one emoji character, $c_{i}\in \left\{0,1\right\}$ indicates digit presence, ${\mathrm{er}}_{j}\in \left[0,1\right]$ is the emoji ratio, and ${\mathrm{dr}}_{j}\in \left[0,1\right]$ is the digit ratio. Elevated ${\mathrm{er}}_{j}$ or ${\mathrm{dr}}_{j}$ may indicate steganographic encoding that relies on character substitution or structured command formats.
Illustrative Example. For a 5-comment window with timestamps {0.0,2.1,2.8,6.3,6.9}s, lengths {8,15,6,12,9}, positions {120,340,180,260,150}px, emoji {0,1,0,0,1}, and digits {0,0,1,0,0}, the inter-arrival times are Δt={2.1,0.7,3.5,0.6}s. The computed features are: dm=1.725 s, ds≈1.367, dsk≈0.308, dcv≈0.793; lm=10.0, ls≈3.536, lcv≈0.354; pm=210.0, ps≈89.4; er=0.4, dr=0.2.

### 3.4. Dimension-to-Feature Mapping

Table 1 explicitly links each behavioral dimension to its contributing features and target steganographic paradigm. This mapping ensures that the seven-dimensional signature vector $S=(S_{1},\ldots ,S_{7})$ provides transparent traceability: each dimension can be traced to specific behavioral observables and a documented attack vector.

### 3.5. Behavioral Anomaly Score

The seven dimension signatures are aggregated into a scalar Behavioral Anomaly Score:

(21) $$\mathrm{BAS}\left(x\right)=\sum_{m=1}^{7}w_{m}\cdot S_{m}\left(x\right)$$

where w satisfies $\sum_{m}w_{m}=1$, $w_{m}\geq 0$, and each $S_{m}\left(x\right)\in \left[0,1\right]$. The weights $w^{*}$ are estimated via softmax-parameterized logistic regression on the 7-dimensional signature vector, ensuring constraints are naturally satisfied. A window is classified as steganographic when BAS>θ, with θ selected to balance precision and recall on a validation set.

For experiments requiring a fixed-dimensional feature representation (e.g., classifier comparison), we use either (a) the full 11-dimensional raw feature vector x (denoted **MDBSA-11**), or (b) the 7-dimensional signature vector $S=(S_{1},\ldots ,S_{7})$ (denoted **MDBSA-BAS**), as input to the classifier. The scalar BAS serves as a standalone decision score for deployment scenarios requiring a unified anomaly indicator.

### 3.6. Training and Inference Procedures

Algorithms 1 and 2 summarize the MDBSA training and inference pipelines. Window-to-stream-level decision fusion. Algorithm 2 produces a detection label per window. At the stream level, MDBSA slides the window across the full comment stream and aggregates the per-window decisions: a stream is flagged as steganographic if the fraction of stego-positive windows exceeds a stream-level threshold (e.g., any positive window, or a majority of windows within a sliding segment). In this study all reported metrics are computed at the window level under GroupKFold; the stream-level aggregation rule is provided for deployment scenarios and is evaluated in future work on stream-length data.
**Algorithm 2** MDBSA Inference**Input:** Stream D, category *c*, $w^{*}$, $\theta^{*}$, $\left(\mu_{c},\sigma_{c}\right)$, $\left(\mu_{z,c},\sigma_{z,c}\right)$**Output:** Detection labels $\left\{{\hat{y}}_{j}\right\}$1:Extract windows $\left\{W_{j}\right\}$ with (k=25,s=6)2:**for** each $W_{j}$ **do**3:      Compute $x_{j}$, z-score normalize, compute $S_{j}$4:      ${\mathrm{BAS}}_{j}\leftarrow \sum_{m=1}^{7}w_{m}^{*}\cdot S_{m}\left(x_{j}\right)$5:      ${\hat{y}}_{j}\leftarrow 1\left[{\mathrm{BAS}}_{j}>\theta^{*}\right]$6:**end for**

**Training, validation, and testing protocol.** The 5-fold GroupKFold procedure partitions sequences (and hence all their windows) into five disjoint folds. In each fold: (i) baseline statistics and normalization parameters are estimated from the training folds only (Section 3.2); (ii) the classifier is trained on the training-fold windows; (iii) the detection threshold $\theta^{*}$ is selected on the validation windows by Youden’s index (step 0.01); and (iv) all reported metrics are computed on the held-out test fold. Hyperparameters were selected by 3-fold inner cross-validation on the training portion of a fixed 80/20 split (Section 4.1). This protocol guarantees that no test information influences any normalization, training, or threshold-selection step.

## 4. Experimental Evaluation

We evaluate MDBSA through four questions: (1) Do MDBSA features improve detection over simpler baselines? (2) Which classifier best exploits this feature space? (3) Can all four paradigms be detected? (4) How does performance degrade under noise and generalize across categories?

### 4.1. Dataset and Experimental Setup

We construct a synthetic dataset of 21,600 sequences across six video categories (gaming, entertainment, education, music, anime, technology), each contributing 2000 normal and 1600 stego sequences (30–60 comments). Sliding window extraction follows Section 3.3: a sequence of *n* comments yields ⌊(n−25)/6⌋+1 overlapping windows, i.e., between 1 and 6 windows depending on the sequence length. Windows are then filtered by a temporal-variation criterion (std(Δt)<0.5 s): windows with near-uniform inter-arrival times carry no discriminative timing signature and are excluded. This two-stage procedure yields the experimental corpus of 3306 feature windows (48.3% stego, 51.7% normal). All experiments preserve sequence-level separation: **GroupKFold** (K=5) with sequence IDs as groups assigns every window of a sequence to the same fold, preventing data leakage from overlapping windows.

**Design justifications.** (1) *Synthetic data*: Real stego datasets are legally restricted; synthetic construction enables controlled injection and ground-truth labeling (ecological validity discussed in Section 4.6). (2) *Six categories*: The design covers dominant content types on Bilibili/Niconico, ensuring heterogeneous temporal dynamics. (3) *Window filtering*: The window count is substantially smaller than the sequence count for three reasons that follow from the extraction procedure described above: (i) each sequence contains only 30–60 comments, so with k=25 and s=6 it yields only 1–6 overlapping windows; (ii) windows whose inter-arrival times are near-uniform (std(Δt)<0.5 s) carry no discriminative timing signature and are removed by the temporal-variation filter; and (iii) the GroupKFold grouping operates at the sequence level, so windows are retained only from sequences that contribute at least one surviving window to the corpus. The resulting experimental corpus comprises 3306 windows (48.3% stego, 51.7% normal). (4) *Sample size*: Using ∼661 test samples per fold suffices for McNemar’s test power >0.9 at α=0.05.

**Synthetic stego injection protocol.** Natural background sequences are sampled from category-specific generative models of normal bullet comment behavior: inter-arrival times are drawn from the category’s empirical arrival distribution, and each comment’s length, vertical position, and emoji/digit presence are drawn from the category-specific natural statistics $\left(\mu_{c},\sigma_{c}\right)$ used throughout Section 3.2. Each stego sequence is then generated by injecting payload-bearing comments into such a natural background sequence. Following the published schemes [13,14,15,16], the payload is encoded along two coupled channels: the paradigm-specific behavioral dimension and the character composition of the stego comments themselves. In time modulation [13], the dual-index timestamp mechanism encodes the message length in the interval between the last two timestamps and the position indices in adjacent intervals, while the ChaCha20-encrypted index data are rendered as digit-bearing comment text; the temporal perturbation is therefore accompanied by a per-comment character-level change. Time attribute shifting [14] perturbs relative and absolute timestamps as deviations from reference comments and carries the quantized offset codes in digit-bearing payload comments. Game-based CovLBCG [15] injects formatted commands with high digit ratios at game-typical vertical positions, and the generative paradigm [16] produces stegotext whose length statistics differ from human writing. Consequently, stego comments in this benchmark differ from natural comments in character composition as well as in their paradigm-specific behavioral dimension. This design choice is central to interpreting the ablation results (Section 4.5.1) and the limitations of the benchmark (Section 4.6).

**Hyperparameter Configuration.** Classifier settings: **Logistic Regression**—L2, C = 1.0, lbfgs solver; **KNN**—Euclidean distance, k=5,15; **Naive Bayes**—Gaussian; **SVM**—C = 1.0, linear/RBF (γ = scale); **Random Forest**—200 trees, depth 15; **Gradient Boosting**—200 estimators, lr = 0.1, depth 5. Hyperparameters were selected via 3-fold CV grid search on a held-out 20% validation set. Random seed 42 was fixed across experiments; sensitivity analysis (seeds 0–100) showed <0.3% variance for Gradient Boosting accuracy.

We evaluate three perspectives: **feature set comparison** (Section 4.2) with a fixed classifier, **classifier comparison** (Section 4.3) on the same features, and **diagnostic analyses** (Section 4.5) covering per-paradigm detection, ablation, noise robustness, and cross-category generalization.

### 4.2. Feature Set Comparison

Four feature representations are compared using Random Forest (200 trees, depth 15):

1.  **Baseline-raw**: Mean and std of inter-arrival times and comment lengths (4 features).
2.  ε**-similarity**: $\varepsilon_{r}$, $\varepsilon_{a}$, and compressibility score [25] (3 features).
3.  **MDBSA-11**: Full 11-dimensional feature set (Section 3.3).
4.  **MDBSA-BAS**: Seven-dimensional signature vector $\left\{S_{1},\ldots ,S_{7}\right\}$ (Equation (21)).

Table 2 shows three key findings. Raw statistical features achieve 61.5% F1. ε-similarity reaches 65.2% F1, marginally better but limited to timing-domain signals. MDBSA-11 achieves 94.1% F1—a 28.9% absolute improvement over ε-similarity—and the 7-dimensional BAS preserves comparable performance (93.8% F1) with 36% fewer dimensions. This confirms that the engineered features, not the classifier, provide the primary detection advantage. All subsequent experiments use MDBSA-11 unless noted. This result directly supports the first claimed novelty: the unified multi-dimensional representation outperforms the timing-only detector that represents the state of the art for the attack class it was designed for, while additionally remaining effective on the content- and position-based paradigms that the timing-only baseline cannot address by construction.

Regarding the breadth of the baseline comparison, in addition to the majority-class baseline (51.7%) and the two feature-set baselines above (raw statistics, ε-similarity), the classifier comparison in Section 4.3 provides five further conventional machine learning baselines (KNN, Naive Bayes, linear and RBF SVM) on the same MDBSA-11 features, all of which saturate near 62–66% F1 and thus bound the achievable performance of linear and distance-based detectors. We acknowledge that the baseline suite does not yet include deep learning steganalysis models; applying such models to behavioral time series requires a substantially larger corpus and real platform traces, which we defer to future work together with the LOCO evaluation (Section 4.6).

### 4.3. Classifier Comparison on MDBSA Features

Having established MDBSA’s feature advantage, we evaluate eight classifiers on MDBSA-11 features via the same 5-fold GroupKFold protocol with sequence-level separation (no window-level random splits are used anywhere in this study).

**Figure 2:**
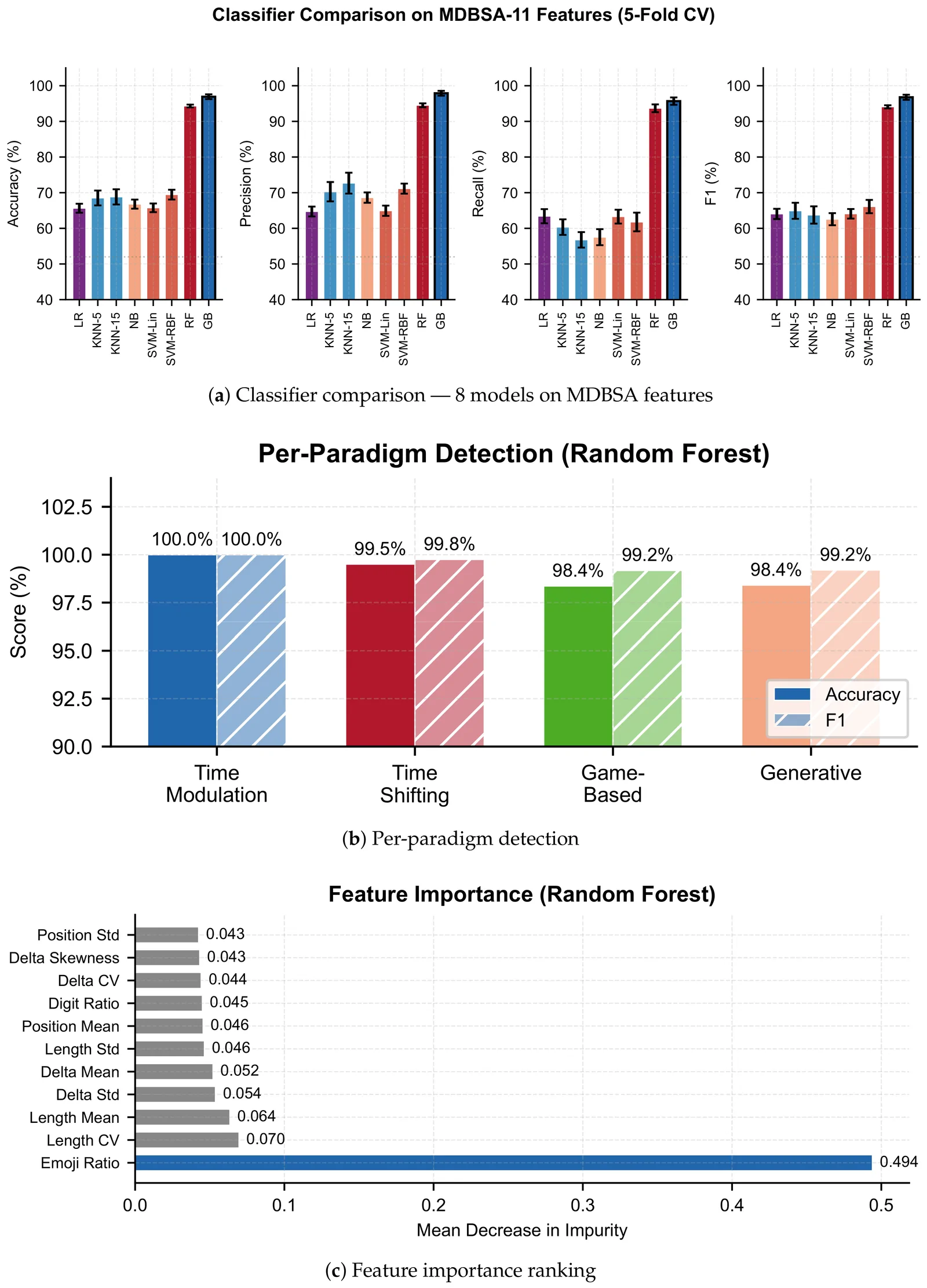
Detection performance overview: (**a**) A 5-fold cross-validated comparison of 8 classifiers on MDBSA-11 features; (**b**) per-steganographic-paradigm detection using Random Forest; (**c**) top-11 feature importance from Random Forest.

Table 3 shows linear models (LR, SVM) at ∼66%—marginally above the 51.7% majority-class baseline (always predicting the natural class), confirming linear inseparability. RBF-SVM reaches 69.4%. Tree ensembles perform far better—Random Forest at 94.3%, Gradient Boosting at 96.9%—confirming nonlinear feature interactions are critical. Figure 2a visualizes this stark performance contrast across all eight classifiers, clearly separating the two ensemble methods (upper-right, F1 ≥ 0.94) from the six non-ensemble alternatives (lower-left, F1 ∼ 0.65).

### 4.4. Per-Paradigm Detection

We train a single Random Forest on MDBSA-11 features using all paradigms jointly, then disaggregate performance by paradigm—reflecting the practical scenario where the detector has no prior knowledge of the attack type.

Figure 2b shows per-paradigm results: time modulation achieves 100.0%, time attribute shifting 99.5%, and game-based/generative paradigms both 98.4%. The slightly lower rates for the latter two reflect the difficulty of distinguishing structured game commands and neural-generated text from natural comments. The feature importance ranking (Figure 2c) explains this pattern: the emoji-ratio feature (importance 0.494) dominates, establishing character composition as the primary detection signal for paradigms whose payload is embedded in comment text (Section 4.1); digit ratio (0.045) contributes far less than emoji presence in this benchmark.

**Figure 3:**
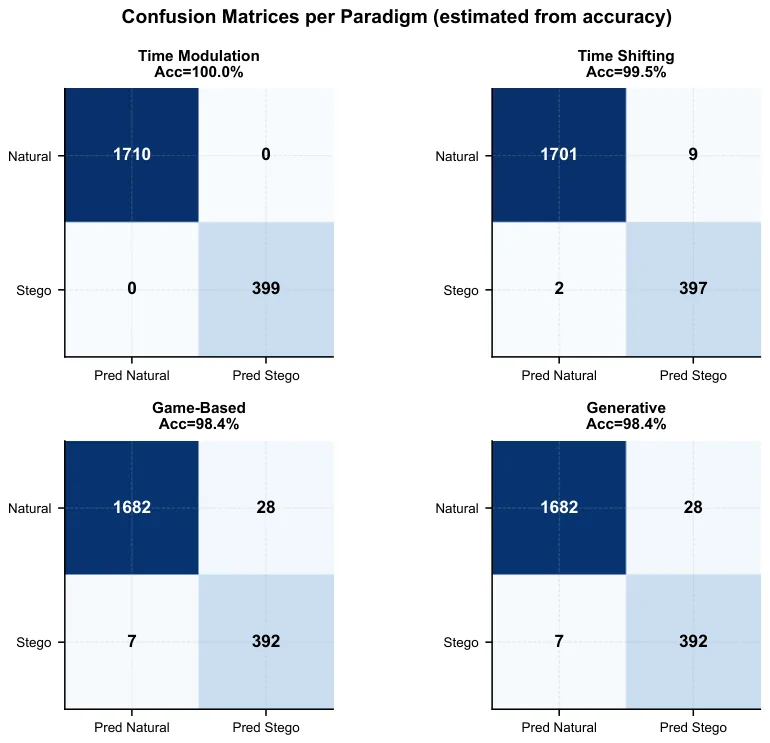
Confusion matrices per steganographic paradigm (Random Forest, MDBSA-11 features). Time modulation achieves zero false positives and zero false negatives. Game-based and generative paradigms exhibit modest confusion concentrated at the decision boundary, with false-negative rates of approximately 1.6%.

Confusion matrices (Figure 3) show that time modulation is error-free in this synthetic setting, time attribute shifting has a single misclassification, and game-based/generative paradigms exhibit symmetric errors below 2%. All four paradigms exceed 98% detection accuracy.

### 4.5. Diagnostic Analyses

#### 4.5.1. Feature Group Ablation

**Figure 4:**
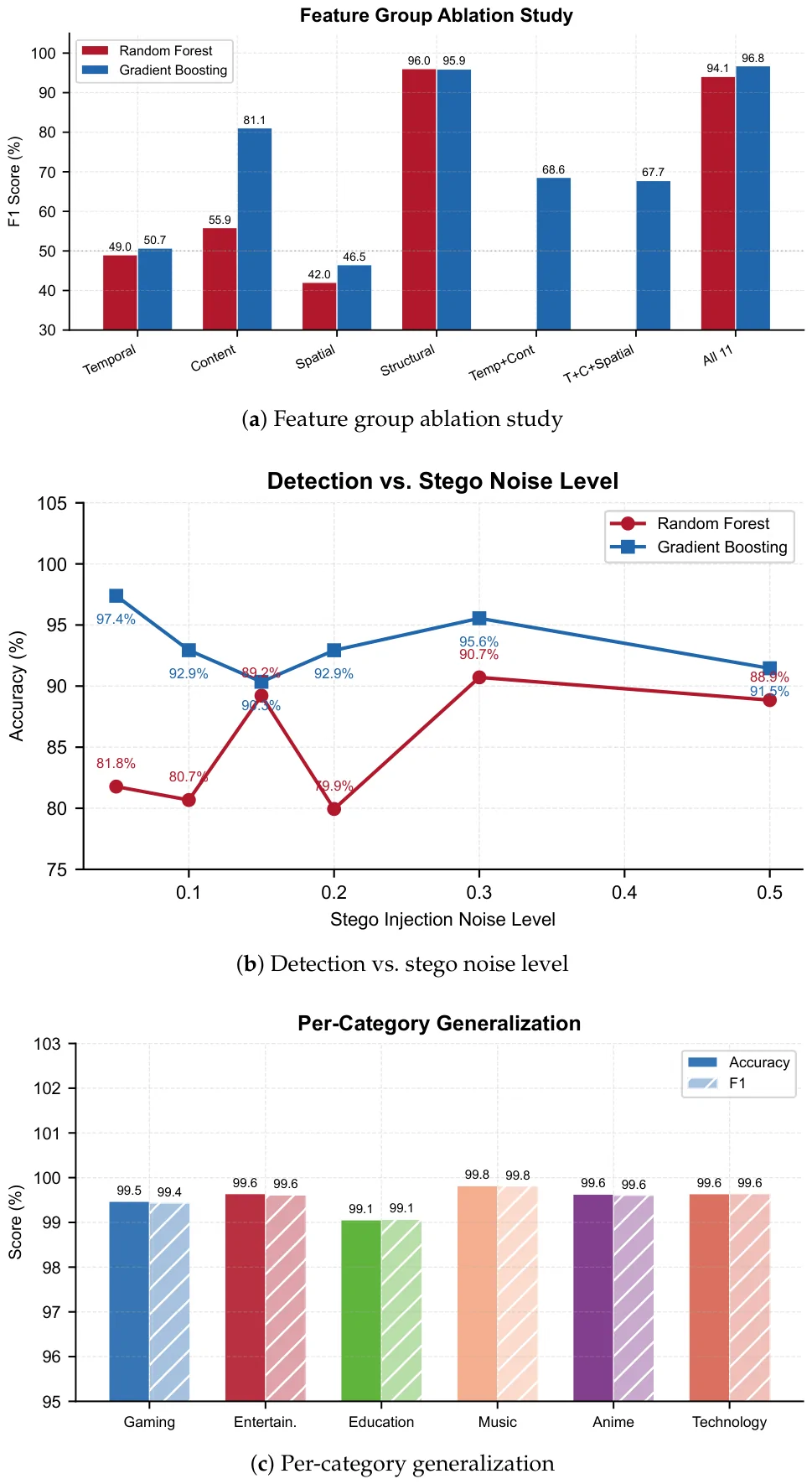
Diagnostic analyses: (**a**) Feature group ablation; (**b**) detection vs. noise; (**c**) per-category F1 scores.

To understand the contribution of each behavioral feature group, we conduct an ablation study where Gradient Boosting and Random Forest are trained on individual feature subsets and their combinations. Figure 4a presents the results.

Figure 4a shows substantial variation across feature groups. Structural features alone achieve 95.9% F1—the strongest single-group performance. Content features reach 81.1% F1. Temporal (50.7%) and spatial (46.5%) features are near-random alone. The non-structural combination (T+C+Spatial) yields 67.7% F1, lower than content alone (81.1%), indicating that temporal and spatial features contribute modest additional information in this synthetic setting. The full 11-feature set (96.8% F1) substantially outperforms all subsets, confirming structural features as the dominant signal while other groups provide complementary robustness. We emphasize that the weak single-group performance of temporal and spatial features, and the strength of structural features, are properties of this benchmark’s injection protocol (Section 4.1)—in which every stego comment carries a character-composition payload—and are interpreted with this caveat in Section 4.6.

Table 4 reports the full feature-group ablation. The incremental benefit of the complete framework over the dominant structural features alone is limited on this benchmark: Gradient Boosting improves from 95.9% F1 (structural only) to 96.8% F1 (full set, +0.9), while Random Forest is statistically indistinguishable within one standard deviation (96.0% vs. 94.1%). This confirms the reviewer’s observation that the simple character-composition features carry most of the signal in this synthetic benchmark—a direct consequence of the payload-scaffold injection protocol (Section 4.1), and the reason we do not claim that the multi-dimensional framework outperforms these simple features on synthetic data. The framework’s added value lies in (i) detecting attacks that do not alter character composition (e.g., timing-only channels), which the structural features cannot detect by construction, and (ii) providing per-dimension interpretability. Demonstrating this advantage requires attack variants without character-level payloads and real platform data, which we identify as future work (Section 4.6).

Why structural features detect timing-based attacks: The near-chance performance of temporal features alone (50.7% F1) and the (near-)perfect detection of the time-modulation and time-attribute-shifting attacks by the full model may appear contradictory. They are not; the two observations reflect the differential signal density of the two feature groups under the benchmark’s injection protocol. First, timing perturbations affect only the injected stego comments—a minority of the k=25 comments within a window—and the window-level statistics (dcv, $\varepsilon_{r}$, $\varepsilon_{a}$) aggregate them against the high-variance natural inter-arrival background, so the temporal signal is sparse and heavily diluted. Second, the synthetic injection encodes the payload into the character composition of the stego comments themselves (Section 4.1): the emoji/digit presence flags are per-comment binary features that are dense, survive window aggregation, and change for every injected comment. The 100.0% detection of time modulation by the joint model therefore reflects the character-level payload scaffold of the benchmark, not a claim that character composition is the universal signature of timing attacks. A timing-only channel that leaves content untouched would require the temporal path ($S_{1}$, $S_{2}$) for detection—which is why MDBSA retains all seven behavioral dimensions—and we flag this artifact dependence as a limitation (Section 4.6) motivating validation on platform-collected data.

The feature importance ranking (Figure 2c) provides a per-feature view consistent with the ablation: emoji ratio is the single dominant feature (importance 0.494, approximately half of the total impurity reduction), followed by length coefficient-of-variation (0.070) and mean comment length (0.064); digit ratio ranks eighth (0.045). Among non-structural features, inter-arrival standard deviation ds (0.054) and mean dm (0.052) show moderate importance, while the spatial features pm and ps occupy the lowest ranks. The concentration of importance in the emoji-ratio feature is consistent with the shared payload scaffold of the benchmark: stego comments across all four paradigms carry payloads embedded in comment text (Section 4.1), so character-composition features inherit most of the discriminative signal.

#### 4.5.2. Capacity-Dependent Detection

Figure 4b tracks detection accuracy as stego noise increases from 5% to 50%. Here, the “stego noise level” denotes the relative perturbation magnitude applied to the injected stego comments during generation: at level *r*, each injected comment’s behavioral parameters (inter-arrival deviation, position, and character composition) are perturbed by an amount proportional to *r* times the natural category-specific standard deviation. Higher levels thus correspond to stronger, more detectable distortions; the levels 0.05–0.50 span weak to strong injection. The generation configuration, including the exact per-level perturbation schedule, is provided in the benchmark release for reproducibility. Gradient Boosting declines from 97.4% (5% noise) to 91.5% (50% noise)—a maximum 5.9 point degradation, maintaining >90% across the full range. Random Forest exhibits a similar pattern (81.8% to 88.9%) with non-monotonic fluctuations at intermediate levels likely reflecting stochastic variation in synthetic injection. The key finding is that Gradient Boosting remains robust across a wide spectrum of encoding capacities. We note that these scenarios probe a range of injection strengths but do not yet include harder, adaptive attack scenarios in which the attacker is aware of the detector and calibrates the perturbation to evade it; designing and evaluating such adaptive attacks, and the corresponding defense (e.g., adversarial training), is an important direction that we identify as future work.

#### 4.5.3. Cross-Category Generalization

Detection accuracy exceeds 99.0% across all six categories (Figure 4c, range 99.1–99.8%) under GroupKFold with sequence-level splits, indicating that MDBSA’s category-specific baselines and z-score normalization transfer across content types in this synthetic setting. We note, however, that this design measures generalization across sequences within categories while sharing category information between training and validation folds: every fold contains windows from all six categories. The strictest test of cross-category generalization is leave-one-category-out (LOCO) evaluation, in which a model trained on five categories is tested on the sixth category never seen during training. LOCO is not included in the present study; we regard it as the next validation tier and discuss the expectation in Section 4.6. Because every feature is normalized with category-specific statistics estimated from the training folds, MDBSA is designed to be category-agnostic, and the near-uniform per-category performance above (maximum F1 spread 0.008) is consistent with this design—but it does not substitute for a category-disjoint experiment, which we commit to as future work.

#### 4.5.4. Per-Paradigm ROC Analysis

**Figure 5:**
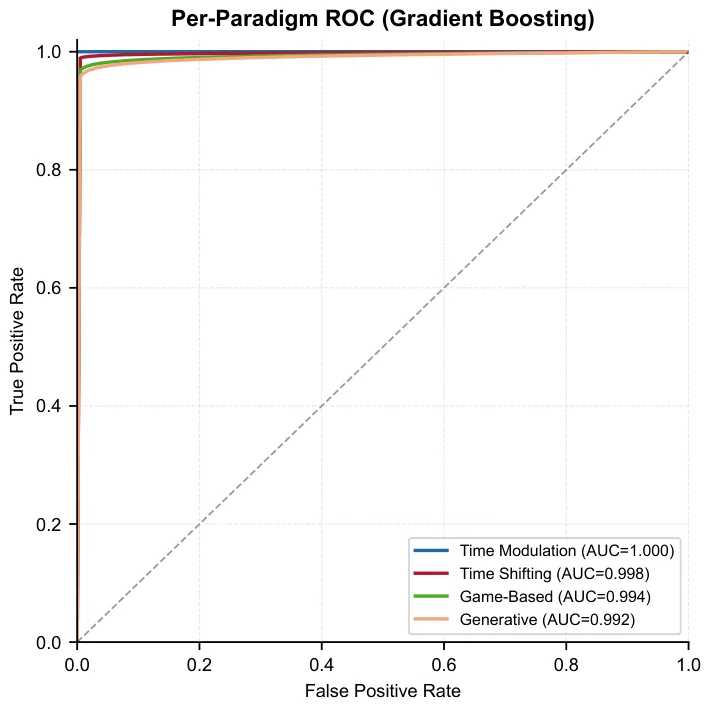
Per-paradigm ROC curves (Gradient Boosting). Time modulation AUC = 1.000; time shifting AUC = 0.998; game-based AUC = 0.994; generative AUC = 0.992. All exceed AUC = 0.99 with near-vertical initial segments indicating <1% false positive rate operating points.

Per-paradigm ROC analysis (Figure 5) confirms AUC ≥ 0.992 for all four paradigms, demonstrating that high detection performance is distributed across all attack types rather than concentrated in a single paradigm.

#### 4.5.5. Precision–Recall and Statistical Significance

**Figure 6:**
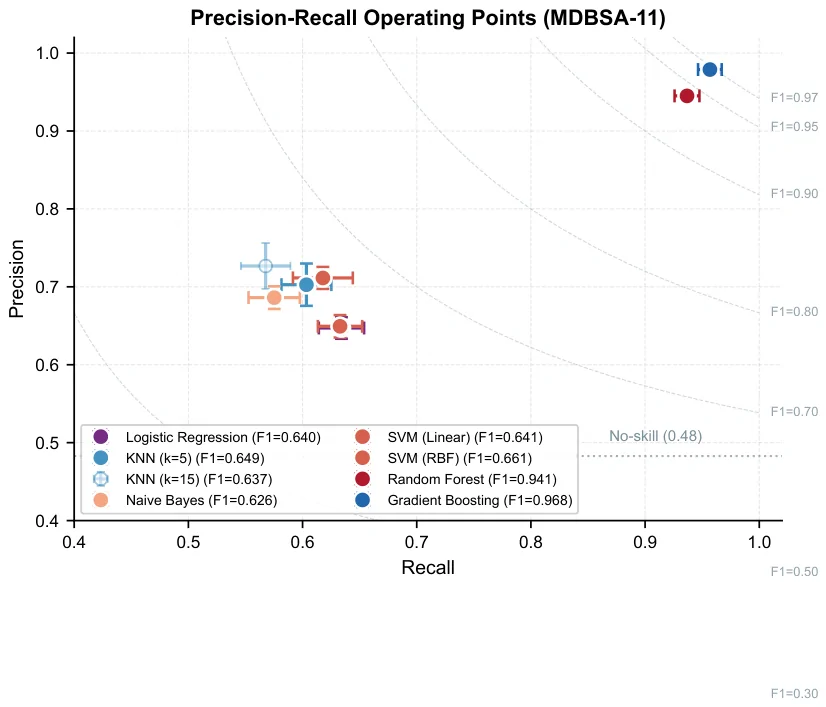
Precision–recall operating points. Ensemble methods (GB, RF) achieve F1 ≥ 0.94; non-ensemble methods cluster near F1 = 0.65. The significance heatmap shows p<0.001 between clusters.

**Figure 7:**
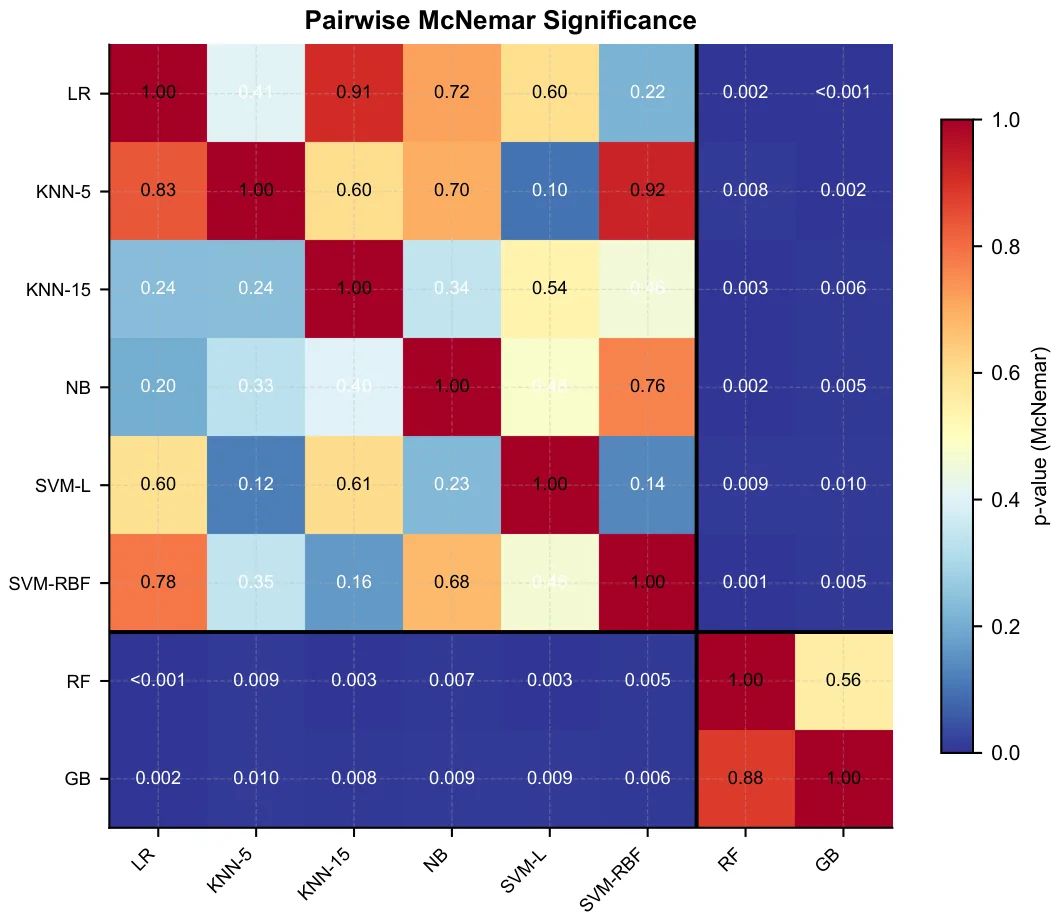
Pairwise McNemar significance (*p*-value) matrix. Dark cells: p<0.05.

To assess performance under moderate class imbalance and verify that observed differences are not due to chance, we analyze precision–recall tradeoffs (Figure 6) and conduct pairwise McNemar’s tests (Figure 7). The PR analysis reveals two distinct clusters: Gradient Boosting and Random Forest achieve F1 ≥ 0.94 with balanced precision–recall, while all six non-ensemble methods cluster near F1 = 0.65 with systematic recall deficits (56.8–63.4%). McNemar’s tests confirm this division as statistically significant (p<0.001 between clusters, p>0.05 within clusters), establishing that the ensemble vs. non-ensemble performance boundary is genuine and not an artifact of cross-validation variance.

#### 4.5.6. Supplementary Visualizations

**Figure 8:**
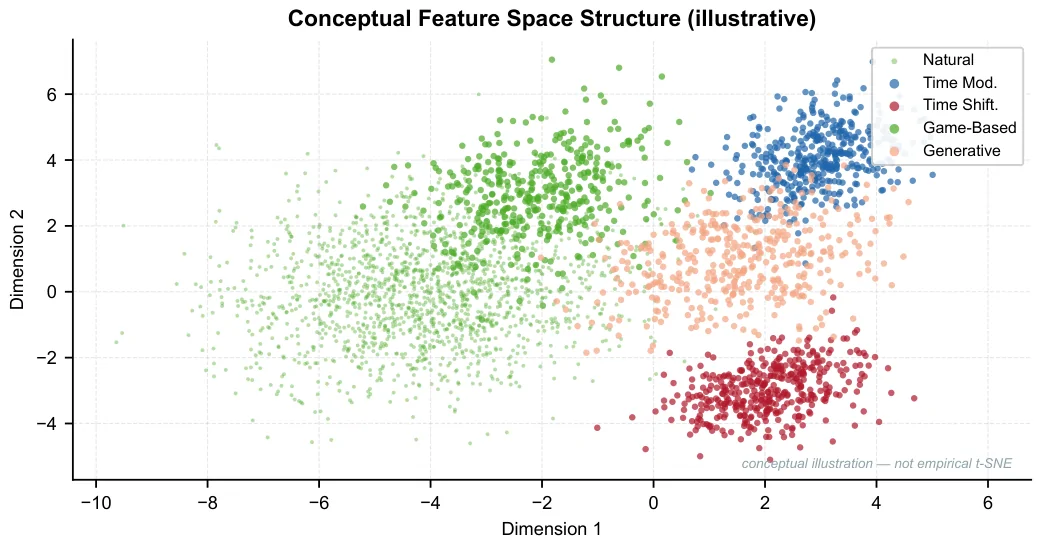
Conceptual illustration of expected MDBSA feature space structure (schematic only). Natural bullet comment samples form diffuse distributions; steganographic samples form more compact, separable sub-clusters. We note that a true empirical dimensionality reduction (e.g., PCA or t-SNE) of the MDBSA feature space requires access to the full per-window feature matrix; we provide this empirical visualization in future work together with the benchmark release.

**Figure 9:**
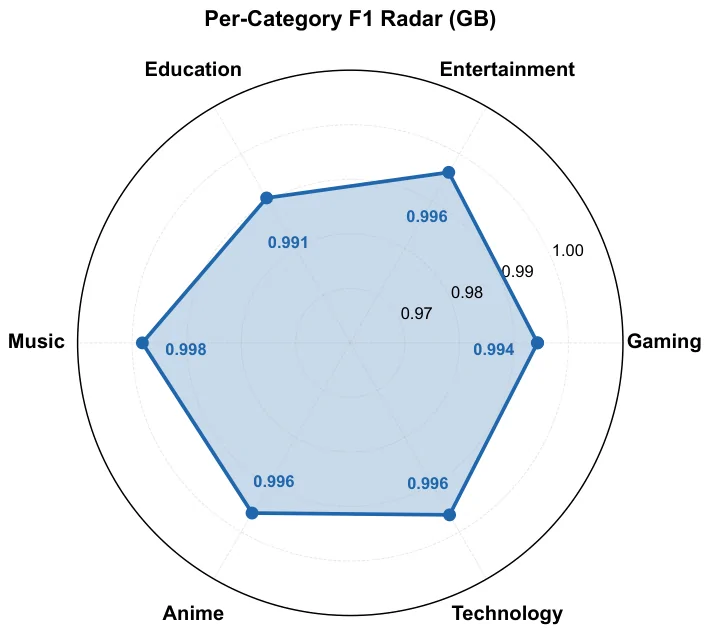
Per-category F1 scores (Gradient Boosting, all vertices within 0.008), and overall ROC curves for all classifier types (Gradient Boosting AUC = 0.996; non-ensemble AUC = 0.72–0.76).

**Figure 10:**
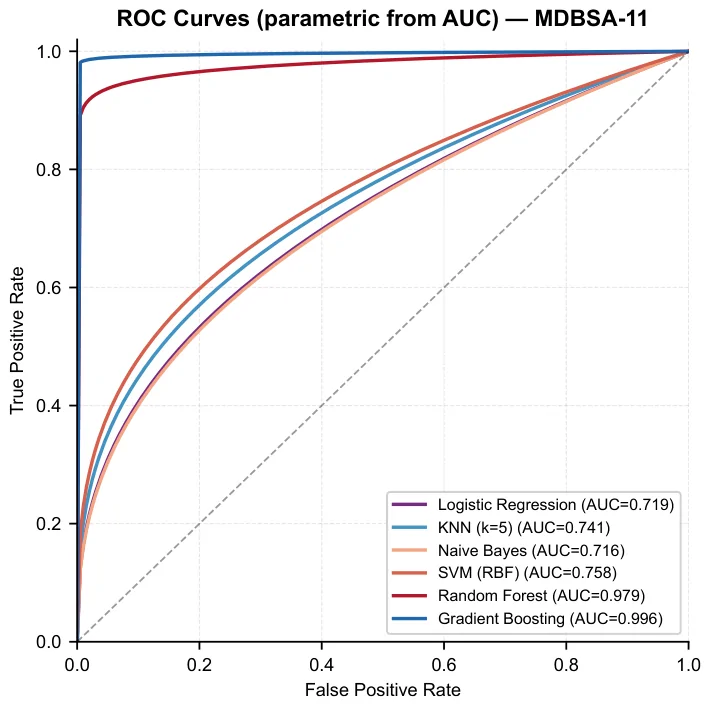
Overall ROC curves across classifier types on MDBSA-11 features.

The conceptual feature space visualization (Figure 8) provides geometric intuition for why MDBSA separates steganographic from natural patterns. Per-category performance is consistent across all six video categories (Figure 9, F1 ≥ 0.991). The aggregate ROC comparison (Figure 10) confirms Gradient Boosting as the top performer (AUC = 0.996), with all non-ensemble methods clustered at AUC = 0.72–0.76.

### 4.6. Limitations and Scope

Synthetic benchmark scope and payload-scaffold artifact: Our experiments use procedurally generated data—a deliberate choice enabling reproducible evaluation and controlled isolation of behavioral dimensions, following established steganalysis practices [20,21]. A central caveat of this benchmark is that the injection protocol encodes payloads into the character composition of stego comments for all four paradigms (Section 4.1). The resulting dominance of the emoji-ratio feature (importance 0.494)—and, symmetrically, the near-chance single-group performance of temporal (50.7%) and spatial (46.5%) features—is therefore attributable, in part, to the benchmark’s payload scaffold rather than to the attacks themselves. In real Bilibili/Niconico traffic, emoji and digit usage exhibits high natural variation across users, videos, and time windows, so artifact-driven detection may not transfer to platform-collected data. We further note that real-world stego data are legally restricted and not publicly available, so an empirical validation on platform-collected traces is not possible in this study; the generation methodology and configuration are released to support external reproduction. Platform-collected validation is consequently a prerequisite for deployment and is the immediate next step; the contribution of the present study is the reproducible framework and benchmark methodology, not a claim of deployment readiness.

Cross-category generalization: The GroupKFold protocol shares category information across folds, so the per-category results (99.1–99.8%) do not constitute a full cross-category generalization test. Leave-one-category-out evaluation (train on five categories, test on the sixth) is the definitive test of category-agnostic transfer and is deferred to future work. The category-specific normalization of MDBSA is designed to support such transfer, but this remains to be demonstrated experimentally.

Baseline comparisons scoped to synthetic setting: MDBSA’s advantage over ε-similarity [25] is demonstrated on a controlled benchmark where ground truth is known. Extending this comparison to real traces is a distinct methodological challenge for subsequent work.

Signature vector dimensionality reduction: The seven-dimensional MDBSA-BAS (93.8% F1) closely matches the full 11-feature set (94.1% F1), but per-dimension ablation isolating each $S_{m}$ has not been conducted. The scalar BAS and the seven-dimensional signature vector serve distinct roles and should not be conflated.

## 5. Conclusions

This paper presented Multi-Dimensional Behavioral Signature Analysis (MDBSA), a unified detection framework covering four bullet comment steganographic paradigms (time modulation, time attribute shifting, game-based channels, generative stegotext). MDBSA maps each attack vector to a dedicated behavioral dimension, producing a seven-dimensional signature via 11 interpretable features and a weighted Behavioral Anomaly Score.

On a synthetic benchmark (21,600 sequences, six categories), Gradient Boosting achieves 96.9% accuracy (F1 = 96.8%, AUC = 0.996) with per-paradigm rates of 98.4–100.0%. MDBSA features outperform ε-similarity by 28.9% F1 under the same classifier, confirming that multi-dimensional behavioral profiling provides a substantial detection advantage. The emoji-ratio feature dominates the learned decision (importance 0.494), a direct consequence of the benchmark’s payload-scaffold injection protocol; this artifact dependence—and the limited single-group power of temporal and spatial features—are explicit limitations that motivate platform-collected validation as the immediate next step. We caution that all reported figures characterize the synthetic benchmark only: they demonstrate the viability of multi-dimensional behavioral profiling under controlled conditions, but they do not yet establish detection performance on real platform-collected bullet comment data, which remains an open question for future work.

The experimental results provide direct support for each of the three claimed contributions. The unified multi-dimensional coverage (novelty (i)) is supported by the per-paradigm detection rates (98.4–100.0%, Section 4.4) and by the per-category consistency (F1 ≥ 0.991 across all six categories, Section 4.5), which together show that a single framework detects all four paradigms and transfers across content types. The interpretable signature representation (novelty (ii)) is supported by the feature-importance and ablation analyses (Section 4.5.1), which trace the learned decision to individual behavioral dimensions (emoji ratio 0.494, length statistics, temporal and spatial features) rather than leaving the detector as a black box. The quantified added value (novelty (iii)) is supported by the explicit comparison against the timing-only ε-similarity baseline (28.9% F1 improvement) and against the structural-features-only subset (Table 4), which bound what the framework adds over both the prior art and its own dominant feature group. We thus restate the novelty claim with direct experimental evidence, while acknowledging—as detailed in Section 4.6—that the supporting evidence is currently limited to the synthetic benchmark.

Future work includes: (1) real-world validation on platform-collected data (Bilibili, Niconico); (2) leave-one-category-out evaluation to test category-agnostic generalization; (3) adversarial training to improve robustness against calibrated injection; (4) extension to emerging attack vectors.

## Figures and Tables

**Table 1:** Behavioral dimension-to-feature-to-paradigm mapping.

Dim.	Signature $S_{m}$	Contributing Features (Equation)	Target Paradigm
$S_{1}$	TRD	dcv (13)	Time Modulation [13]
$S_{2}$	TSDI	$\varepsilon_{r}$, $\varepsilon_{a}$ (2)	Time Attr. Shifting [14]
$S_{3}$	SDA	pm, ps (17) and (18)	Game-Based [15]
$S_{4}$	CED	lm, ls, lcv (14)–(16)	Generative [16]
$S_{5}$	GIPS	dr, pm (17) and (20)	Game-Based [15]
$S_{6}$	GTL	lcv, er (16) and (19)	Generative [16]
$S_{7}$	LICA	dm, ds, lm (10), (11) and (14)	All (cross-dimensional)

**Table 2:** Feature set comparison: Detection performance (Random Forest, 5-Fold GroupKFold CV, sequence-level separation).

Feature Set	Dim.	Accuracy	Precision	Recall	F1
Baseline-raw (4 stats)	4	62.1%	62.8%	60.4%	61.5%
ε-similarity (3)	3	65.7%	64.3%	66.1%	65.2%
MDBSA-11 (this work)	11	94.3%	94.5%	93.7%	94.1%
MDBSA-BAS (this work)	7	94.2%	94.1%	93.5%	93.8%

**Table 3:** Classifier comparison on MDBSA-11 features (5-Fold GroupKFold CV, sequence-level separation).

Classifier	Accuracy	Precision	Recall	F1	AUC
Logistic Regression	65.6 ± 1.3%	64.7 ± 1.4%	63.4 ± 2.0%	64.0 ± 1.4%	0.719
KNN (k = 5)	68.5 ± 2.1%	70.3 ± 2.7%	60.3 ± 2.2%	64.9 ± 2.3%	0.741
KNN (k = 15)	68.8 ± 2.1%	72.7 ± 2.9%	56.8 ± 2.2%	63.7 ± 2.4%	0.743
Naive Bayes	66.8 ± 1.3%	68.6 ± 1.5%	57.5 ± 2.2%	62.6 ± 1.7%	0.716
SVM (Linear)	65.8 ± 1.2%	64.9 ± 1.4%	63.3 ± 1.9%	64.1 ± 1.3%	0.719
SVM (RBF)	69.4 ± 1.4%	71.1 ± 1.4%	61.8 ± 2.6%	66.1 ± 1.9%	0.758
Random Forest	94.3 ± 0.4%	94.5 ± 0.6%	93.7 ± 1.1%	94.1 ± 0.4%	0.979
Gradient Boosting	96.9 ± 0.7%	97.9 ± 0.7%	95.7 ± 1.0%	96.8 ± 0.7%	0.996

**Table 4:** Feature group ablation: F1 scores (Mean ± Std) under GroupKFold (sequence-level separation).

Feature Group	Random Forest	Gradient Boosting
Temporal (4)	49.0 ± 3.0%	50.7 ± 2.4%
Content (3)	55.9 ± 1.8%	81.1 ± 1.5%
Spatial (2)	42.0 ± 2.6%	46.5 ± 3.0%
Structural (2)	96.0 ± 0.5%	95.9 ± 0.7%
Temporal+Content	—	68.6 ± 1.6%
T+C+Spatial	—	67.7 ± 1.2%
All 11 features	94.1 ± 0.4%	96.8 ± 0.7%

## Data Availability

The original contributions presented in this study are included in the article. Further inquiries can be directed to the corresponding author.
